# Inhibition of Lipid Accumulation and Cyclooxygenase-2 Expression in Differentiating 3T3-L1 Preadipocytes by Pazopanib, a Multikinase Inhibitor

**DOI:** 10.3390/ijms22094884

**Published:** 2021-05-05

**Authors:** Anil Kumar Yadav, Byeong-Churl Jang

**Affiliations:** Department of Molecular Medicine, College of Medicine, Keimyung University, 1095 Dalgubeoldaero, Dalseo-gu, Daegu 42601, Korea; aydaegu@gmail.com

**Keywords:** pazopanib, 3T3-L1, C/EBP-α, PPAR-γ, perilipin A, AMPK, COX-2

## Abstract

Pazopanib is a multikinase inhibitor with anti-tumor activity. As of now, the anti-obesity effect and mode of action of pazopanib are unknown. In this study, we investigated the effects of pazopanib on lipid accumulation, lipolysis, and expression of inflammatory cyclooxygenase (COX)-2 in differentiating and differentiated 3T3-L1 cells, a murine preadipocyte. Of note, pazopanib at 10 µM markedly decreased lipid accumulation and triglyceride (TG) content during 3T3-L1 preadipocyte differentiation with no cytotoxicity. Furthermore, pazopanib inhibited not only expression of CCAAT/enhancer-binding protein-α (C/EBP-α), peroxisome proliferator-activated receptor-γ (PPAR-γ), and perilipin A but also phosphorylation of signal transducer and activator of transcription (STAT)-3 during 3T3-L1 preadipocyte differentiation. In addition, pazopanib treatment increased phosphorylation of cAMP-activated protein kinase (AMPK) and its downstream effector ACC during 3T3-L1 preadipocyte differentiation. However, in differentiated 3T3-L1 adipocytes, pazopanib treatment did not stimulate glycerol release and hormone-sensitive lipase (HSL) phosphorylation, hallmarks of lipolysis. Moreover, pazopanib could inhibit tumor necrosis factor (TNF)-α-induced expression of COX-2 in both 3T3-L1 preadipocytes and differentiated cells. In summary, this is the first report that pazopanib has strong anti-adipogenic and anti-inflammatory effects in 3T3-L1 cells, which are mediated through regulation of the expression and phosphorylation of C/EBP-α, PPAR-γ, STAT-3, ACC, perilipin A, AMPK, and COX-2.

## 1. Introduction

Obesity is defined as abnormal fat accumulation in the human body. Obesity has now become a global pandemic, based on the fact that it is highly associated with the development of many human chronic diseases such as type 2 diabetes, hypertension, and cancer [1,2]. A wealth of information illustrates that excessive preadipocyte differentiation leads to excessive fat (mainly in the form of triglyceride (TG)) accumulation in adipocytes and, resultantly, the development of obesity [3,4]. Lipolysis is a biological process through which excessive TG is hydrolyzed into glycerol and free fatty acids in differentiated (mature) adipocytes [5] and is regarded as a therapeutic regime against obesity and related diseases [6]. Thus, any substance that decreases lipid accumulation and increases lipolysis in adipocytes can be considered as a potential anti-obesity agent.

Preadipocyte differentiation (adipogenesis) is a multistep process that occurs in cellular, morphological, and biochemical changes. The process converts fibroblast-like preadipocytes into differentiated (or mature) adipocytes that are filled with many lipid droplets (LDs) [7,8]. Multiple adipogenic transcription factors, including CCAAT/enhancer-binding proteins (C/EBPs), peroxisome proliferator-activated receptors (PPARs), and signal transducer and activator of transcription (STAT) proteins, play a pivotal role in preadipocyte differentiation [9,10]. Preadipocyte differentiation also involves lipogenesis and LD maturation/stabilization, which requires fatty acid synthase (FAS), acetyl-CoA carboxylase (ACC), and perilipin A [11,12,13]. Increasing evidence further indicates that several protein kinases, including cAMP-activated protein kinase (AMPK), protein kinase A (PKA), extracellular signal-regulated protein kinase-1/2 (ERK-1/2), p38 mitogen-activated protein kinase (MAPK), and protein kinase C (PKC), participate in the regulation of preadipocyte differentiation [14,15,16,17]. In differentiated adipocytes, lipolysis is controlled by hormone-sensitive lipase (HSL), PKA, AMPK, and ERK-1/2 [5,18,19].

Adipocytes in the adipose tissues (ATs) can be exposed to various types of internal and external stimuli, such as pro-inflammatory cytokines, free fatty acids, or lipopolysaccharide (LPS), and they are thought to express and secrete many inflammatory factors, ultimately leading to the development of obesity inflammation [20,21,22]. Tumor necrosis factor (TNF)-α is a pro-inflammatory cytokine that stimulates (pre)adipocytes to express and secrete inflammatory mediators and chemokines [23], thereby exacerbating inflammation and recruiting macrophages in the ATs [24]. Cyclooxygenase (COX), responsible for the biosynthesis of prostaglandins (PGs), exists in two isoforms: COX-1, the constitutive form that has beneficial functions such as water resorption and stomach protection, and COX-2, the inducible form that involves inflammation and pain [25]. It is documented that TNF-α is a strong inducer of COX-2 in various cells, including (pre)adipocytes [26,27,28,29,30]. Thus, inhibition (or an inhibitor) of TNF-α-induced COX-2 expression in (pre)adipocytes is considered as a potential target in alleviating obesity inflammation.

Drug repositioning is defined as the investigation of existing drugs for new therapeutic purposes [31,32]. We have recently tested the modulatory effects of 86 protein kinase inhibitors on lipid accumulation during the differentiation of 3T3-L1 preadipocytes into adipocytes. Of note, pazopanib was found to have an inhibitory effect on lipid accumulation in 3T3-L1 preadipocyte differentiation. Pazopanib is an oral multikinase inhibitor with anticancer activity [33,34,35]. Its antitumor effect is thought to be exerted through selective inhibition of vascular endothelial growth factor receptor (VEGFR)-mediated angiogenesis, as well as its direct blockade of growth-promoting receptor tyrosine kinases (RTKs), including platelet-derived growth factor receptors (PDGFRs), fibroblast growth factor receptors (FGFRs), and KIT [35,36,37]. However, the anti-obesity effect and mechanism of action of pazopanib remain unknown. In this study, we investigated the regulatory effects of pazopanib on lipid accumulation, lipolysis, and expression of TNF-α-induced COX-2 in 3T3-L1 cells. We here demonstrate that pazopanib has strong anti-adipogenic and anti-inflammatory, but not lipolytic, effects on 3T3-L1 cells, which are mediated through control of the expression and phosphorylation of C/EBP-α, PPAR-γ, STAT-3, ACC, perilipin A, AMPK, and COX-2.

## 2. Results

### 2.1. Pazopanib (10 µM) Markedly Reduces Lipid Accumulation and TG Content in Differentiating 3T3-L1 Preadipocytes with No Significant Cytotoxicity

**Figure 1:**
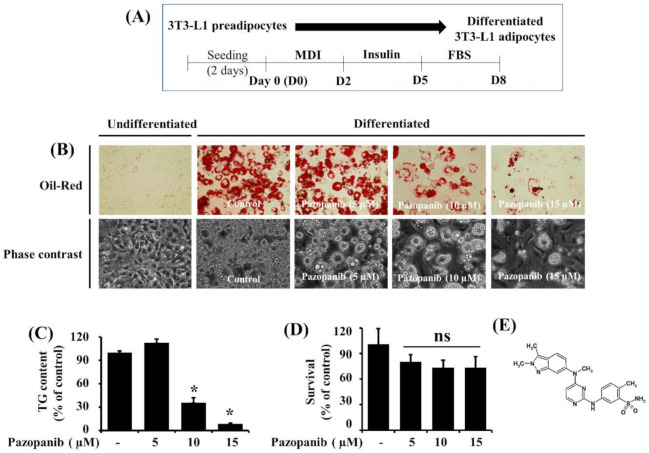
Effects of pazopanib on lipid accumulation, TG content, and cell growth (survival) during 3T3-L1 preadipocyte differentiation. (**A**) The experimental scheme for 3T3-L1 preadipocyte differentiation. (**B**) Measurement of intracellular lipid droplets (LDs) accumulation in undifferentiated 3T3-L1 preadipocytes on day 0 (D0) or differentiated adipocytes on day 8 (D8) in the absence (control; 0.1% DMSO) or presence of pazopanib at the designated concentrations by Oil Red O staining (upper panels) and by phase-contrast image (lower panels) at 40× (**C**) Measurement of intracellular TG content in control or pazopanib-treated 3T3-L1 cells on D8 by AdipoRed assay. Data are mean ± SE of three independent experiments, each done in triplicate. * *p* < 0.05 vs. control. (**D**) Measurement of the number of live cells in control or pazopanib-treated 3T3-L1 cells on D8 by cell count assay. Data are mean ± SE of three independent experiments, each done in triplicate. (**E**) The chemical structure of pazopanib.

The experimental scheme for 3T3-L1 preadipocyte differentiation is shown in Figure 1A. Whether pazopanib could curb lipid accumulation during 3T3-L1 preadipocyte differentiation was primarily investigated using Oil Red O staining. In the absence of pazopanib, there was a high accumulation of lipid droplets (LDs) in 3T3-L1 cells on D8 of differentiation compared with undifferentiated cells at D0 (Figure 1B, upper panels). Of interest, pazopanib treatment concentration-dependently suppressed accumulation of LDs in 3T3-L1 cells on D8 of differentiation. Pazopanib’s suppressive effect on LD accumulation in 3T3-L1 cells on D8 of differentiation was also confirmed by phase-contrast microscopy (Figure 1B, lower panels). Subsequent AdipoRed assay revealed that pazopanib at 10 or 15 µM significantly reduced intracellular TG content in 3T3-L1 cells on D8 of differentiation (Figure 1C). Data of cell count analysis further showed that pazopanib up to 15 μM was not cytotoxic to 3T3-L1 cells on D8 of differentiation (Figure 1D). The chemical structure of pazopanib is presented in Figure 1E. Due to strong reductive effects on LD accumulation and TG content with no significant cytotoxicity, we chose the 10 µM concentration of pazopanib for further studies.

### 2.2. Pazopanib Strongly Reduces Expression and Phosphorylation Levels of Adipogenic Transcription Factors C/EBP-A, PPAR-Γ, and STAT-3 in Differentiating 3T3-L1 Preadipocytes

**Figure 2:**
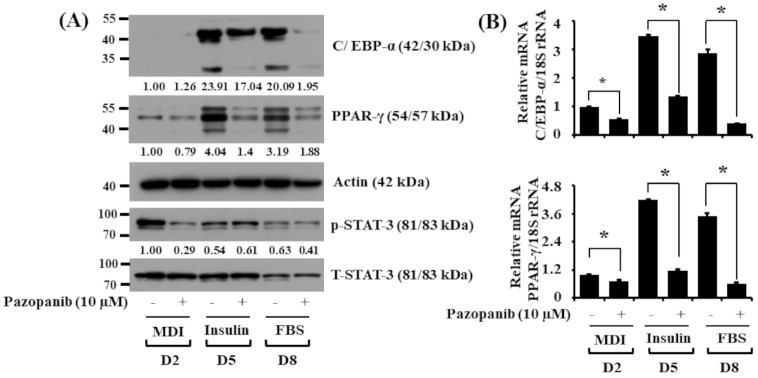
Effects of pazopanib on expression and phosphorylation levels of C/EBP-α, PPAR-γ, and STAT-3 during 3T3-L1 preadipocyte differentiation. (**A**) 3T3-L1 preadipocytes were differentiated with induction medium containing MDI, insulin, and FBS in the absence (control; 0.1% DMSO) or presence of pazopanib (10 µM), and harvested at day 2 (D2), D5, and D8, respectively. At each time point, whole-cell lysates were prepared and analyzed by immunoblot analysis with respective antibodies. p-STAT-3, phosphorylated STAT-3; T-STAT-3, total STAT-3. (**B**) 3T3-L1 preadipocytes were differentiated with induction medium containing MDI, insulin, and FBS in the absence or presence of pazopanib (10 µM), and harvested at D2, D5, and D8, respectively. At each time point, total cellular RNA was extracted and analyzed by real-time qPCR with respective primers. mRNA levels of C/EBP-α and PPAR-γ were normalized to those of control 18S rRNA. Data are mean ± SE of three independent experiments, each done in duplication. * *p* < 0.05 vs. control.

To define molecular mechanisms underlying pazopanib’s lipid- effects, we next examined the effects of pazopanib (10 µM) on expression and phosphorylation levels of major adipogenic transcription factors, such as C/EBP-α, PPAR-γ, and STAT-3, during the differentiation of 3T3-L1 preadipocytes into adipocytes using immunoblot analysis. As shown in Figure 2A, pazopanib at 10 µM greatly decreased protein expression levels of C/EBP-α and PPAR-γ in 3T3-L1 cells on D5 and D8 of differentiation. Moreover, pazopanib markedly reduced phosphorylation levels of STAT-3 on D2 of differentiation. Protein expression levels of total STAT-3 and control actin remained unchanged at the times tested. We next performed real-time qPCR to investigate the effects of pazopanib (10 µM) on C/EBP-α and PPAR-γ mRNA expressions during 3T3-L1 preadipocyte differentiation. As shown in Figure 2B, pazopanib at 10 µM largely decreased transcripts of C/EBP-α and PPAR-γ in 3T3-L1 cells on D2, D5, and D8 of differentiation.

### 2.3. Pazopanib Alters Expression and Phosphorylation Levels of FAS, ACC, Perilipin A, AMPK, Leptin, and Resistin in Differentiating 3T3 Preadipocytes

**Figure 3:**
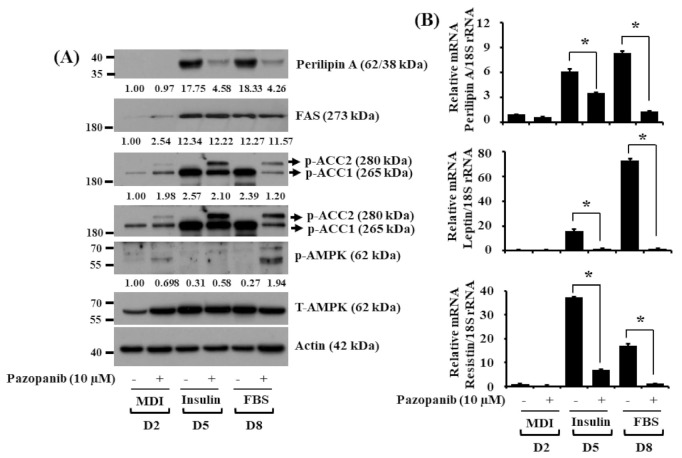
Effects of pazopanib on expression and phosphorylation levels of perilipin A, FAS, ACC, AMPK, leptin, and resistin during 3T3-L1 preadipocyte differentiation. (**A**) 3T3-L1 preadipocytes were differentiated with induction medium containing MDI, insulin, and FBS in the absence (control; 0.1% DMSO) or presence of pazopanib (10 µM), and harvested at day 2 (D2), D5, and D8, respectively. At each time point, whole-cell lysates were prepared and analyzed by immunoblot analysis with respective antibodies. p-ACC, phosphorylated ACC; T-ACC, total ACC; p-AMPK, phosphorylated AMPK; T-AMPK, total AMPK. (**B**) 3T3-L1 preadipocytes were differentiated with induction medium containing MDI, insulin, and FBS in the absence or presence of pazopanib (10 µM), and harvested at D2, D5, and D8, respectively. At each time point, total cellular RNA was extracted and analyzed by real-time qPCR with respective primers. mRNA levels of perilipin A, leptin, and resistin were normalized to those of control 18S rRNA. Data are mean ± SE of three independent experiments, each done in duplication. * *p* < 0.05 vs. control.

We next determined whether pazopanib (10 µM) modulates expression and phosphorylation levels of other adipogenesis regulatory proteins and enzymes, such as perilipin A, FAS, ACC, and AMPK, during 3T3-L1 preadipocyte differentiation. As shown in Figure 3A, pazopanib at 10 µM strongly reduced protein expression levels of perilipin A in 3T3-L1 cells on D5 and D8 of differentiation. Distinctly, while pazopanib did not affect protein expression levels of FAS, it largely increased phosphorylation levels of ACC on D2, D5, and D8. Notably, pazopanib elevated AMPK phosphorylation without affecting its total protein levels on D8. Total expression levels of control actin protein remained constant under these experimental conditions. Data of real-time qPCR further demonstrated that pazopanib significantly down-regulated transcripts of not only perilipin A but also leptin and resistin, two adipokines, in 3T3-L1 cells on D5 and D8 of differentiation (Figure 3B).

### 2.4. Pazopanib Does Not Stimulate Glycerol Release and HSL Phosphorylation in Differentiated 3T3-L1 Cells

**Figure 4:**
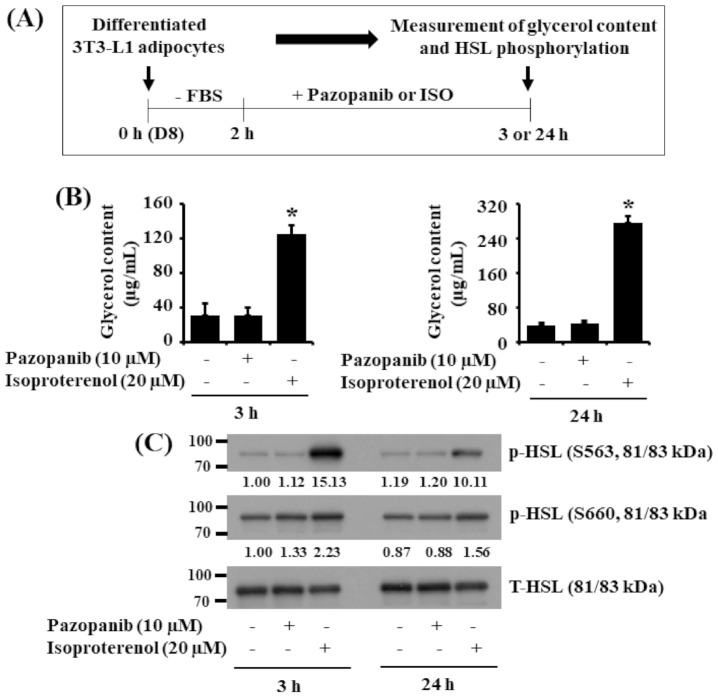
Effects of pazopanib on glycerol release and HSL phosphorylation in differentiated 3T3-L1 cells. (**A**) The experimental scheme for measurement of glycerol content and HSL phosphorylation in differentiated 3T3-L1 cells. (**B**) Differentiated 3T3-L1 cells on D8 (0 h) were serum-starved for 2 h and then grown in the absence (control; 0.1% DMSO) or presence of pazopanib (10 µM) or ISO (20 µM) for additional 3 and 24 h, respectively. Glycerol content in culture medium from control or drug (pazopanib or ISO)-treated cells was measured in triplicate. Data are mean ± SE of three independent experiments. * *p* < 0.05 vs. control. (**C**) Differentiated 3T3-L1 cells on D8 (0 h) were serum-starved for 2 h and then grown in the absence or presence of pazopanib (10 µM) or ISO (10 µM) for an additional 3 and 24 h, respectively. At each time point, whole-cell lysates were prepared and analyzed by immunoblot analysis with respective antibodies. p-HSL, phosphorylated HSL; T-HSL, total HSL.

We next investigated whether pazopanib (10 μM) induces lipolysis in differentiated 3T3-L1 cells. Pazopanib’s lipolytic effect was herein assessed by the drug’s ability to increase glycerol release and HSL phosphorylation on serine (S) residues S563 and S660. In comparison, isoproterenol (ISO), a known lipolytic agent [38], at 20 μM was used as a positive control. The experimental scheme for measurement of glycerol content and HSL phosphorylation is depicted in Figure 4A. As anticipated, ISO treatment for 3 or 24 h produced high glycerol content in the culture media of differentiated 3T3-L1 cells (Figure 4B). By contrast, pazopanib at 10 μM did not elevate it at the times tested. Furthermore, while ISO treatment highly increased HSL S563 and S660 phosphorylation, pazopanib treatment had no or little effect on it in differentiated 3T3-L1 cells (Figure 4C). Expression levels of total HSL proteins remained unchanged under these experimental conditions.

### 2.5. Pazopanib Strongly Inhibits TNF-A-Induced COX-2 Protein and mRAN Expressions in Both 3T3-L1 Preadipocytes and Differentiated Cells

**Figure 5:**
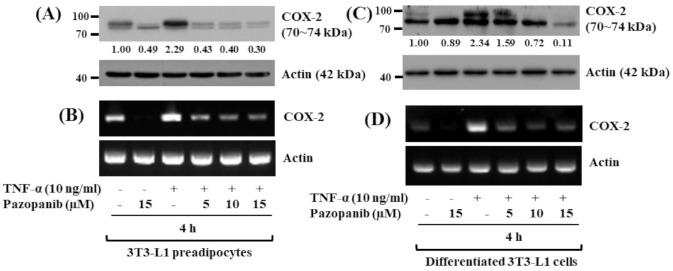
Effects of pazopanib on TNF-α-induced COX-2 expression in 3T3-L1 preadipocytes and differentiated cells. (**A**,**B**) 3T3-L1 preadipocytes were treated without or with TNF-α (10 ng/mL) in the presence or absence of pazopanib at the designated concentrations for 4 h. Whole-cell lysate and total RNA were prepared and analysed by Western blotting (**A**) and RT-PCR (**B**), respectively. (**C**,**D**) Differentiated 3T3-L1 cells on D8 were treated without or with TNF-α (10 ng/mL) in the presence or absence of pazopanib at the indicated doses for 4 h. Whole-cell lysate and total RNA were prepared and analysed by Western blotting (**C**) and RT-PCR (**D**), respectively.

In order to see any anti-inflammatory effect, we next sought to explore whether TNF-α up-regulates COX-2 protein and mRNA expressions in 3T3-L1 cells, and pazopanib inhibits it. Strikingly, as shown in Figure 5A,B, while treatment with TNF-α at 10 ng/mL for 4 h highly induced expression of COX-2 at both protein and mRNA levels in 3T3-L1 preadipocytes, pazopanib treatment concentration-dependently suppressed it. In differentiated 3T3-L1 cells, TNF-α at 10 ng/mL for 4 h was able to substantially induce COX-2 protein and mRNA expressions. However, pazopanib treatment also inhibited the cytokine-induced COX-2 protein and mRNA expressions in differentiated 3T3-L1 cells in a dose-dependent manner (Figure 5C,D).

## 3. Discussion

Pazopanib is a multiple kinase inhibitor that blocks tumor growth and inhibits vascular neogenesis [33,34,35], presenting its anti-cancer effect. Until now, pazopanib’s anti-obesity effect and mode of action have been unknown. Here we report that pazopanib at 10 µM has anti-adipogenic and anti-inflammatory, but not lipolytic, effects on 3T3-L1 cells, and these effects are mediated through control of the expression and phosphorylation levels of C/EBP-α, PPAR-γ, STAT-3, perilipin A, ACC, AMPK, and COX-2.

In initial experiments, we demonstrated that pazopanib at 10 µM has strong anti-adipogenic/anti-lipogenic effects, given that it largely reduces lipid accumulation and TG content in differentiating 3T3-L1 cells with no cytotoxicity. There is a wealth of information supporting a pivotal role of C/EBP-α, PPAR-γ, and STAT-3 transcription factors in preadipocyte differentiation [9,10]. To date, pazopanib regulation of C/EBP-α, PPAR-γ, and STAT-3 in adipocytes was unknown. In the current study, pazopanib greatly down-regulated C/EBP-α and PPAR-γ at both protein and mRNA levels in differentiating 3T3-L1 cells, showing that pazopanib inhibits C/EBP-α and PPAR-γ expression through their transcriptional repression. Moreover, we showed pazopanib’s ability to greatly inhibit STAT-3 phosphorylation without affecting its total protein expression levels on D2, an early stage of differentiation. These results indicate that pazopanib inhibits phosphorylation of pre-existing STAT-3 without de novo protein synthesis. Given that STAT-3 can be activated at an early stage of 3T3-L1 preadipocyte differentiation [39], which is crucial for C/EBP-α and PPAR-γ transcriptional up-regulation at a middle or late stage of differentiation [40], it is conceivable that pazopanib-mediated reduction of C/EBP-α and PPAR-γ expression in differentiating 3T3-L1 cells is in part due to early STAT-3 inhibition. These results collectively suggest that pazopanib’s anti-adipogenic/lipogenic effect on differentiating 3T3-L1 cells is largely associated with the drug’s ability to reduce expression and phosphorylation of these adipogenic transcription factors.

The aforementioned adipocyte differentiation also involves lipogenesis and LD stabilization processes [4,8]. FAS and ACC are key lipogenic enzymes responsible for the synthesis of fatty acids [11,12]. Perilipin A is a protein that binds to and stabilizes the newly synthesized LDs [13,39,41,42], which is thus important for lipid storage and accumulation during adipocyte differentiation. At present, little is known about pazopanib regulation of FAS, ACC, and perilipin A in adipocytes. We here showed that pazopanib strongly reduces cellular levels of perilipin A in differentiating 3T3-L1 cells, suggesting destabilization of newly formed LDs, which results in blockage of adipogenesis. Moreover, the present study illustrates that pazopanib does not affect cellular levels of FAS, but it highly elevates levels of phosphorylated ACC, which are inactive forms of ACC [43], in differentiating 3T3-L1 cells. Inactivation of ACC leads to an increase of lipid β-oxidation [44]. These results show that perilipin A down-regulation and ACC inhibition may further contribute to pazopanib’s anti-adipogenic/lipogenic effect.

AMPK is a sensor of cellular energy status that plays an essential role in the regulation of cellular energy homeostasis [14]. Of note, studies have previously shown that activation of AMPK inhibits 3T3-L1 preadipocyte differentiation [45,46,47]. To date, little is known about pazopanib regulation of AMPK in adipocytes. In the current study, we demonstrated that pazopanib induces AMPK phosphorylation on T172, which is an active form of AMPK [14], in differentiating 3T3-L1 cells, supporting pazopanib-induced AMPK activation. Accordingly, activation of AMPK leads to phosphorylation of multiple downstream targets including ACC [48]. It is thus likely that pazopanib induces activation of AMPK, which phosphorylates (inhibits) ACC in differentiating 3T3-L1 cells, which may be a part of pazopanib’s anti-adipogenic/lipogenic effect. At present, how pazopanib induces AMPK activation in differentiating 3T3-L1 cells remains unclear. AMPK is regarded as a fat controller of the energy railroad because it has a central role in controlling the synthesis of fatty acids, triglycerides, and cholesterol as well as the oxidation of fatty acids [49]. There is further evidence that activation of AMPK inhibits ATP-consuming anabolic processes while it activates ATP-producing catabolic processes [43,49], supporting the role of AMPK in the regulation of lipid metabolism in response to metabolic stress and energy demand. AMPK is activated by increases in the cellular ATP/AMP ratio caused by metabolic stresses that either interfere with ATP production or that accelerate ATP consumption [50]. Given that pazopanib increases AMPK phosphorylation but decreases ACC phosphorylation in differentiating 3T3-L1 cells, it is likely that the drug-induced AMPK activation in differentiating 3T3-L1 cells is associated with metabolic stress due to reduction of cellular ATP content and energy demand to accelerate ATP synthesis. It has been previously demonstrated that AMPK is activated and phosphorylated by several upstream kinases including liver kinase B1 (LKB1) and Ca^2+^/calmodulin-dependent protein kinase (CaMKK) [51]. LKB-1, a serine/threonine kinase, is a tumor suppressor gene, which is ubiquitously expressed in mammalian cells and is activated in a complex with two scaffolding proteins: STE20-related adaptor (STRAD) protein and mouse protein 25 (MO25) [52]. This complex directly activates AMPK by phosphorylating Thr172 of the α subunit. [52]. Moreover, LKB1 functions as a master upstream kinase activator for another 12 kinases that are related to AMPK, including the salt-inducible kinases (SIKs) [53]. In addition to LKB1, CaMKK, Ca^2+^/calmodulin-dependent protein kinase, has also been shown to phosphorylate and activate AMPK in response to increases in intracellular Ca^2+^ including 3T3-L1 [51]. It will be interesting to see, in the future, whether pazopanib affects the expression and phosphorylation of LKB1 and CaMKK and alters the ATP/AMP ratio in 3T3-L1 differentiating cells.

It is documented that adipocytes express and secrete an array of adipokines including leptin and resistin, which are involved in the pathogenesis of obesity and type 2 diabetes [54,55,56,57]. In this study, of note, pazopanib treatment markedly reduced transcript levels of leptin and resistin during 3T3-L1 preadipocyte differentiation. These results further advocate that pazopanib could be an important lead for the medication of obesity and related disorders in which overproduction of leptin and resistin is problematic.

Accumulating evidence supports obesity as a chronic inflammatory disease [58]. It is known that (pre)adipocytes, a predominant cell type present in the adipose tissues, express and secrete a variety of adipokines (leptin, adiponectin, etc.) as well as inflammatory cytokines (TNF-α, IL-1β, IL-6, etc.) and enzymes (iNOS, COX-2, etc.) [23], which confer obesity inflammation. We and others have previously demonstrated TNF-α-induced expression of COX-2 in 3T3-L1 (pre)adipocytes [26,27,28,29,30]. However, to date, little is known about pazopanib regulation of TNF-α-induced expression of COX-2 in 3T3-L1 preadipocytes and differentiated cells. In the present study, of interest, we found that treatment with pazopanib at 5–15 μM for 4 h largely inhibits TNF-α-induced expression of COX-2 in both 3T3-L1 preadipocytes and differentiated cells. It is documented that inflammation confers expansion of the white adipose tissues by increasing adipogenesis, though the primary event triggering this is not fully understood [59]. It is assumed that acute and chronic expression of inflammatory mediators in the white adipose tissues and (pre)adipocytes partly leads to the development of obesity [58]. These results thus advocate that pazopanib’s anti-inflammatory effect on 3T3-L1 preadipocytes and differentiated 3T3-L1 cells by down-regulating COX-2 may further contribute to the drug’s anti-obesity effect. However, it should be noted that pazopanib’s anti-adipogenic/lipogenic and anti-inflammatory effects herein are seen in cultured 3T3-L1 cells. Future studies are therefore warranted to investigate whether pazopanib could inhibit lipid accumulation and inflammation in obese animal models.

In conclusion, this is the first report demonstrating that pazopanib has strong anti-adipogenic/lipogenic and anti-inflammatory effects on 3T3-L1 cells, and these effects are mediated through control of the expression and phosphorylation levels of C/EBP-α, PPAR-γ, STAT-3, ACC, perilipin A, AMPK, and COX-2. This work presents drug repositioning of pazopanib and its potential therapeutics for the treatment of obesity.

## 4. Materials and Methods

### 4.1. Chemicals and Antibodies

Pazopanib was purchased from Selleckchem (Houston, TX, USA). 3-Isobutyl-1-methylxanthine (IBMX), dexamethasone, insulin, and Oil Red O Stock Solution were bought from Sigma (St. Louis, MO, USA). Enhanced chemiluminescence (ECL) reagents were bought from Advansta (San Jose, CA, USA). A detailed list of antibodies used in this study is included in Supplementary Table mentary Table S1.

### 4.2. Cell Culture and Differentiation

3T3-L1 preadipocytes (ATCC, Manassas, VA, USA) were cultured in Dulbecco’s Modified Eagle’s Medium (DMEM, Welgene, Daegu, Korea) supplemented with 10% heat-inactivated fetal calf serum (FCS, Gibco, Grand Island, NY, USA) and 1% penicillin/streptomycin (Welgene, Daegu, South Korea) at 37 °C in a humidified atmosphere of 5% CO_2_. 3T3-L1 preadipocytes were grown up to the contact inhibition stage and remained in the post-confluent stage for 2 days (D0). Differentiation was induced by changing the medium to DMEM supplemented with 10% FBS (Welgene, Daegu) plus a cocktail of hormones (MDI): 0.5 mM IBMX (M), 0.5 µM dexamethasone (D), and 5 µg/mL insulin (I) for 2 days (D2). The cells were then switched to DMEM supplemented with 10% FBS and 5 µg/mL insulin for an additional 3 days (D5). The cells were fed every other day with DMEM supplemented with 10% FBS for an additional 3 days (D8). In some experiments, the media was also supplemented at all time-points with different concentrations (5, 10, and 15 μM) of pazopanib.

### 4.3. Oil Red O Staining

Oil Red O working solution was made by mixing 6 mL of 0.1% Oil Red O Stock Solution in isopropanol and 4 mL of double-distilled water. On D8 of differentiation, control or pazopanib-treated 3T3-L1 cells were washed with phosphate-buffered saline (PBS), fixed with 10% formaldehyde for 2 h at room temperature (RT), washed with 60% isopropanol, and dried. The fixed cells were then stained with Oil Red O working solution for 1 h at RT and washed with deionized water. Stained LDs in control or pazopanib-treated 3T3-L1 cells were visualized under the light microscope (Nikon, TS100, Tokyo, Japan).

### 4.4. Cell Count Assay

3T3-L1 preadipocytes were seeded in a 24-well plate and differentiated as above. On D8 of differentiation, control or pazopanib-treated 3T3-L1 cells were stained with trypan blue dye, and those that excluded the dye were counted using a light microscope. The cell count assay was done in triplicates and repeated three times.

### 4.5. Quantification of Intracellular Triglyceride (TG) Content by Adipored Assay

On D8 of differentiation, intracellular TG content in control or pazopanib-treated 3T3-L1 cells was measured with AdipoRed assay reagent kit in consonance with the manufacturer’s instructions (Lonza, Basel, Switzerland). Fluorescence intensity was quantified with excitation and emission at 485 and 572 nm, respectively, using Victor3 (Perkin Elmer, Waltham, MA, USA).

### 4.6. Quantification of Glycerol Content

Differentiated 3T3-L1 adipocytes were serum-starved for 2 h and incubated with pazopanib (10 µM) or isoproterenol (ISO, 20 µM), a known lipolysis inducer, for 3 h and 24 h, respectively. At each time point, the culture medium was saved, and glycerol content was measured by a free glycerol reagent (Sigma, St. Louis, MO, USA) according to the manufacturer’s instructions. Absorbance was measured at a wavelength of 540 nm using the microplate reader.

### 4.7. Preparation of Whole-Cell Lysates

3T3-L1 cells were washed with PBS and lysed in a modified radioimmunoprecipitation assay buffer (Sigma) at designated time points. The cell lysates were collected and centrifuged at 12,074× *g* for 20 min at 4 °C. The supernatant was saved, and its protein concentration was determined using bicinchoninic acid (BCA) protein assay kit (Thermo Scientific, Rockford, IL, USA).

### 4.8. Immunoblot Analysis

Proteins (50 µg) were separated by SDS-polyacrylamide gel electrophoresis (SDS-PAGE) and transferred onto nitrocellulose membranes (Millipore, Bedford, MA, USA). The membranes were washed with Tris-buffered saline (TBS) (10 mM Tris-Cl, 150 mM NaCl, pH 7.5) supplemented with 0.05% (*v*/*v*) Tween 20 (TBST) followed by blocking with TBST containing 5% (*w*/*v*) non-fat dried milk. The membranes were incubated with specific antibodies at 4 °C. The membranes were rinsed with TBST and further incubated with a horseradish peroxidase-conjugated secondary antibody (anti-goat IgG, anti-mouse IgG, or anti-rabbit IgG) for 2 h at RT. The membranes were then rinsed three times with TBST and developed with ECL reagents. β-Actin expression levels were used to control for equal protein loading.

### 4.9. Quantitative Real-Time RT-PCR

Total cellular RNA was isolated from control or pazopanib-treated 3T3-L1 cells using RNAiso Plus (TaKaRa, Kusatsu, Shiga, Japan). Three µg of total RNA was used to prepare complementary DNA using a random hexadeoxynucleotide primer and reverse transcriptase. SYBR green (TaKaRa, Kusatsu, Shiga, Japan) was used to quantitatively determine mRNA levels of genes with LightCycler ^®^96 Machine (Roche, Mannheim, Germany). PCR reactions were run in duplicate for each sample, and mRNA levels of each gene were normalized to the levels of 18S ribosomal RNA (rRNA). Primer sequences used in this study are listed in Table S2.

### 4.10. Reverse-Transcription Polymerase Chain Reaction (RT-PCR)

Three micrograms of total RNA were transcribed in the same way as in Section 4.9. The cDNA prepared above was amplified by PCR with the primers listed in Table S3. Levels of actin mRNA expression were used as an internal control.

### 4.11. Statistical Analyses

Data are expressed as mean ± standard error (SE) of the mean. One-way ANOVA followed by Dunnett’s post hoc test was performed using SPSS 11.5 software (SPSS, Inc., Armonk, NY, USA). *p* < 0.05 was considered to indicate statistically significant differences.

## Data Availability

Data is contained within the article.

## Supplementary Materials

The following are available online at https://www.mdpi.com/article/10.3390/ijms22094884/s1.
