# Supplementary material for: Inhibition of Lipid Accumulation and Cyclooxygenase-2 Expression in Differentiating 3T3-L1 Preadipocytes by Pazopanib, a Multikinase Inhibitor
Source: Int J Mol Sci. 2021 May 5;22(9):4884. doi: 10.3390/ijms22094884 (PMC8125232; doi:10.3390/ijms22094884)
Supplement: Supplementary file 1 [file ijms-22-04884-s001.zip › ijms-1181636-supplementary.pdf]

Supplementary Table S1. List of antibodies used for Western blot analysis.

Antibodies	Dilution Used	Source	Catalog No.
Primary Antibodies			
C/EBP-α	1:2,000	Santa Cruz Biotechnology	sc-61
PPAR-γ	1:2,000	Santa Cruz Biotechnology	sc-7272
p-STAT-3 (Y705)	1:2,000	Santa Cruz Biotechnology	sc-8059
STAT-3	1:2,000	Santa Cruz Biotechnology	sc-8019
Perilipin A	1:2,000	BioVision	#3948-200
FAS	1:2,000	BD Bioscience	#9452
p-AMPK (T172)	1:2,000	Cell signalling	#2535
AMPK	1:2,000	Cell signalling	#2793
p-ACC (S79)	1:2,000	Cell signalling	#3661
ACC	1:2,000	Cell signalling	#3662
p-HSL (S563)	1:2,000	Cell signalling	#4139
p-HSL (S660)	1:2,000	Cell signalling	#4126
HSL	1:2,000	Cayman chemical	#10006371
COX-2	1:2,000	Cayman chemical	#160106
β-Actin	1:10,000	Sigma	A5441
Secondary Antibodies			
Goat anti-rabbit IgG-HRP	1:5,000	Jackson ImmunoResearch Laboratories	111-035-045
Goat anti-mouse-IgG-HRP	1:5,000	Jackson ImmunoResearch Laboratories	115-035-062

Supplementary Table S2. Sequences of primers used for quantitative real-time PCR.

Gene	Forward	Reverse
C/EBP-α	TTACAACAGGCCAGGTTTCC	CTCTGGGATGGATCGATTGT
PPAR-γ	GGTGAAACTCTGGGAGATTC	CAACCATTGGGTCAGCTCTC
FAS	TTGCTGGCACTACAGAATGC	AACAGCCTCAGAGCGACAAT
Perilipin A	CTTTCTCGACACACCATGGAAAC	CCACGTTATCCGTAACACCCTTCA
Leptin	CCAAAACCCTCATCAAGACC	CTCAAAGCCACCACCTCTGT
Resistin	CCGATGAGCAGTCA CCTCCA	CAGCTGCTTCGCCTCGTCCTCCT
18S rRNA	GGTGAAGGTCGGTGTGAACG	GGTAGGAACACGGAAGGCCA

Supplementary Table S3. Sequences of primers used for reverse-transcription polymerase chain reaction (RT-PCR).

Gene	Forward	Reverse
COX-2	TTGAAGACCAGGAGTACAGC	GGTACAGTTCCATGACATCG
Actin	TCATGAAGTGTGACGTT-GACATCCGT	CCTAGAAGCATTTGCGGTGCAC-GATG
